# Deep neuromuscular block reduces the incidence of intra-operative complications during laparoscopic donor nephrectomy: a pooled analysis of randomized controlled trials

**DOI:** 10.1186/s13741-021-00224-1

**Published:** 2021-12-09

**Authors:** Gabby T. J. A. Reijnders-Boerboom, Esmee V. van Helden, Robert C. Minnee, Kim I. Albers, Moira H. D. Bruintjes, Albert Dahan, Chris H. Martini, Frank C. H. d’Ancona, Gert-Jan Scheffer, Christiaan Keijzer, Michiel C. Warlé

**Affiliations:** 1 Department of Surgery, Radboud University Medical Centre, Geert Grooteplein Zuid 10, 6525 GA Nijmegen, The Netherlands; 2 Department of Anaesthesiology, Radboud University Medical Centre, Geert Grooteplein Zuid 10, 6525 GA Nijmegen, The Netherlands; 3 Department of Surgery, Erasmus Medical Centre, Doctor Molewaterplein 40, 3015 GD Rotterdam, The Netherlands; 4 Department of Anaesthesiology, Leiden University Medical Centre, Albinusdreef 2, 2333 ZA Leiden, The Netherlands; 5 Department of Urology, Radboud University Medical Centre, Geert Grooteplein Zuid 10, 6525 GA Nijmegen, The Netherlands

**Keywords:** Intra-operative complications, Postoperative complications, Laparoscopy, Artificial pneumoperitoneum, Neuromuscular blockade

## Abstract

**Study objective:**

To assess whether different intensities of intra-abdominal pressure and deep neuromuscular blockade influence the risk of intra-operative surgical complications during laparoscopic donor nephrectomy.

**Design:**

A pooled analysis of ten previously performed prospective randomized controlled trials.

**Setting:**

Laparoscopic donor nephrectomy performed in four academic hospitals in the Netherlands: Radboudumc, Leiden UMC, Erasmus MC Rotterdam, and Amsterdam UMC.

**Patients:**

Five hundred fifty-six patients undergoing a transperitoneal, fully laparoscopic donor nephrectomy enrolled in ten prospective, randomized controlled trials conducted in the Netherlands from 2001 to 2017.

**Interventions:**

Moderate (tetanic count of four > 1) versus deep (post-tetanic count 1–5) neuromuscular blockade and standard (≥10 mmHg) versus low (<10 mmHg) intra-abdominal pressure.

**Measurements:**

The primary endpoint is the number of intra-operative surgical complications defined as any deviation from the ideal intra-operative course occurring between skin incision and closure with five severity grades, according to ClassIntra. Multiple logistic regression analyses were used to identify predictors of intra- and postoperative complications.

**Main results:**

In 53/556 (9.5%) patients, an intra-operative complication with ClassIntra grade ≥ 2 occurred. Multiple logistic regression analyses showed standard intra-abdominal pressure (*OR* 0.318, *95% CI* 0.118–0.862; *p* = 0.024) as a predictor of less intra-operative complications and moderate neuromuscular blockade (*OR* 3.518, *95% CI* 1.244–9.948; *p* = 0.018) as a predictor of more intra-operative complications. Postoperative complications occurred in 31/556 (6.8%), without significant predictors in multiple logistic regression analyses.

**Conclusions:**

Our data indicate that the use of deep neuromuscular blockade could increase safety during laparoscopic donor nephrectomy. Future randomized clinical trials should be performed to confirm this and to pursue whether it also applies to other types of laparoscopic surgery.

**Trial registration:**

Clinicaltrials.gov LEOPARD-2 (NCT02146417), LEOPARD-3 trial (NCT02602964), and RELAX-1 study (NCT02838134), Klop et al. (NTR 3096), Dols et al. 2014 (NTR1433).

## Introduction

The use of deep neuromuscular blockade (NMB) defined as a post-tetanic count (PTC) of 1–2(Albers et al., 2019) may facilitate laparoscopic surgery for two reasons. First, surgical space conditions as rated by surgeons on a subjective scale from 1 to 5(Torensma et al., 2016) are significantly better in patients undergoing laparoscopic surgery with a deep NMB, as compared to a moderate NMB.(Bruintjes et al., 2017) The main component of surgical condition rating scale is the intra-abdominal working space. A few clinical studies indicate that the intra-abdominal working space is increased by using deep instead of moderate NMB.(Lindekaer et al., 2013; Van Wijk et al., 2015; Madsen et al., 2015) Secondly, deep NMB provides better surgical stillness as compared to moderate NMB (TOF 1–2)(Blobner et al., 2015), as on average the diaphragm is fully relaxed with a deep but not with a moderate block.(Pansard et al., 1987) Therefore, the use of deep NMB during laparoscopy may either enable the use of lower insufflation pressures or improve surgical conditions at standard intra-abdominal pressure. The use of low intra-abdominal pressure (IAP) (<10 mmHg) during laparoscopic surgery reduces abdominal pain, referred shoulder pain, and analgesic consumption as compared to standard pressure (≥10 mmHg).(Warlé et al., 2013; Gurusamy et al., 2009; Özdemir-van Brunschot et al., 2016; Singla & Mittal, 2014) Nevertheless, the use of low IAP is still under debate because it remains unclear if improved patient recovery outweighs a theoretically increased risk of intra-operative surgical complications related to limited working space. To assess whether different intensities of IAP and NMB influence the risk of intra-operative surgical complications, we performed a secondary analysis on ten randomized controlled trials in adults undergoing transperitoneal, fully laparoscopic donor nephrectomy.

## Material and methods

### Ethics

Approval for the four randomized controlled trials initiated by Radboudumc was given by the Central Committee on Research involving Human Subjects of the Radboud University Nijmegen Medical Center.(Warlé et al., 2013; Özdemir-van Brunschot et al., 2017; Özdemir-van Brunschot et al., 2018; Bruintjes et al., 2019) Approval for the five randomized controlled trials initiated by Erasmus MC Rotterdam was given by the Institutional Review Board of the Erasmus MC in Rotterdam.(Klop et al., 2014; Dols et al., 2014; Dols et al., 2010; Kok et al., 2006a; Kok et al., 2006b) One trial initiated by Amsterdam UMC was approved by the Medical Ethical Committee of the Academic Medical Center Amsterdam.(Minnee et al., 2008) Oral and written informed consent was obtained from all patients before inclusion.

### Pooled analysis

We performed a secondary analysis on pooled data (*n* = 556) of ten previously conducted prospective, double-blinded randomized controlled trials (Table 1). The methods and primary outcomes of these trials(Warlé et al., 2013; Özdemir-van Brunschot et al., 2017; Özdemir-van Brunschot et al., 2018; Bruintjes et al., 2019; Klop et al., 2014; Dols et al., 2014; Dols et al., 2010; Minnee et al., 2008; Kok et al., 2006a; Kok et al., 2006b) in laparoscopic donor nephrectomy patients were previously published. All trials were initiated by Radboudumc, Erasmus MC Rotterdam, and Amsterdam UMC and performed in four academic teaching hospitals in the Netherlands from 2001 to 2017. Anesthesia and surgery were protocolized and similar, except for the depth of neuromuscular blockade, intra-abdominal pressure, and analgesia. Baseline characteristics such as type of anesthesia, gender, ASA classification, age, and body mass index (BMI) are presented in Table 1.

**Table 1:** Trial overview and baseline characteristics

	Participating centers	Recruitment period	Number of patients	IAP (mmHg)	Type of NMB^**b**^	Type of anesthesia	Gender ***N*** (%)		ASA classification ***N*** (%)			Age Mean (SD)	BMI Mean (SD)
							Male	Female	1	2	3		
Warle et al. (2013)	RUMC	2011–2012	20	7 vs 14	TOF 1–2	TIVA	10 (50.0%)	10 (50.0%)	17 (85%)	3 (15%)	0 (0%)	51.15 (±9.33)	25.32 (±3.21)
Ozdemir-Van Brunschot et al. (2017)	RUMC	2014–2015	64	6 vs 12	PTC 1–5	Volatile	36 (56.3%)	28 (43.8%)	44 (69%)	20 (31%)	0 (0%)	54.94 (±12.04)	25.92 (±3.19)
Ozdemir-Van Brunschot et al. (2018)	RUMC	2015–2016	34	6	TOF 1–2 vs PTC 1–5	Volatile	22 (64.7%)	23 (35.3%)	N/A	N/A	N/A	50.19 (±12.49)	25.54 (±3.74)
Bruintjes et al. (2019)	RUMC LUMC	2016–2017	96	12	“Single-dose” vs PTC 1–2	TIVA	45 (46.9%)	51 (53.1%)	61 (64%)	35 (36%)	0 (0%)	56.14 (±9.90)	26.46 (±2.99)
Klop et al. (2014)	EMCR	2011–2012	20^a^	14	“Single-dose”	TIVA	5 (25.0%)	15 (75.0%)	14 (70%)	5 (25%)	1 (5%)	49.72 (±14.39)	25.09 (±3.19)
Dols et al. (2014)	EMCR	2008–2010	95^a^	14	“Single-dose”	TIVA	39 (41.1%)	56 (58.9%)	59 (62%)	35 (37%)	1 (1%)	51.59 (±12.99)	25.96 (±4.13)
Dols et al. (2010)	EMCR RUMC	2006–2008	40	12	“Single-dose”	TIVA	24 (60.0%)	16 (40.0%)	29 (73%)	11 (28%)	0 (0%)	53.41 (±9.62)	26.94 (±3.78)
Minnee et al. (2008)	AUMC	2002–2006	105	12	“Single-dose”	TIVA	44 (41.9%)	61 (58.1%)	85 (81%)	20 (19%)	0 (0%)	47.73 (±11.86)	25.61 (±3.57)
Kok et al. (2006a, b)	EMCR	2001–2004	49^a^	12	“Single-dose”	TIVA	25 (51.0%)	24 (49.0%)	39 (80%)	10 (20%)	0 (0%)	49.40 (±14.73)	25.73 (±3.57)
Kok et al. (2006a, b)	EMCR RUMC	2001–2004	50^a^	12	“Single-dose”	TIVA	18 (52.9%)	16 (47.1%)	N/A	N/A	N/A	47.31 (±13.23)	26.13 (±4.45)

*RUMC* Radboud University Medical Centre, *LUMC* Leiden University Medical Centre, *EMCR* Erasmus Medical Centre Rotterdam, *AUMC* Amsterdam University Medical Centre, *TIVA* total intravenous anesthesia, *IAP* intra-abdominal pressure, *NMB* neuromuscular blockade, *ASA* American Society of Anesthesiologists classification system, *BMI* body mass index

^a^Number of patients who underwent laparoscopic donor nephrectomy (transperitoneal approach)

^b^All studies used rocuronium as neuromuscular blocking agent

### Intra-operative complications

The primary outcome measure was the number of intra-operative complications with a severity score of two or higher, according to the validated Classification of Intraoperative Complications (ClassIntra) score.(Kaafarani & Velmahos, 2015; Rosenthal et al., 2015; Kinaci et al., 2016; Dell-Kuster et al., 2015; Dell-Kuster et al., 2020) This classification is a recently, well-validated classification system for intra-operative complications, featuring simple but inclusive definitions. The classification includes five severity grades depending on the need for treatment and degree of life-threat: with grade 1, a complication without symptoms and no need for treatment; grade 2, a complication with moderate symptoms and the need for additional treatment; grade 3, a complication with severe symptoms, potentially life-threatening, and the need for moderate additional treatment; grade 4, a complication with life-threatening symptoms and the need for major or urgent treatment; and grade 5, being fatal, leading to intra-operative death.(Dell-Kuster et al., 2020) In all studies, grade ≥ 2 intra-operative complications were recorded prospectively.

### Outcomes

Secondary outcome measures included operation time, estimated blood loss, 30-day postoperative complications, and length of hospital admission. Postoperative complications were recorded during the first thirty postoperative days and graded according to the Clavien-Dindo classification.(Dindo et al., 2004; Mitropoulos et al., 2018) The Clavien-Dindo scale varies from grade 1, meaning every deviation from the normal postoperative course without the need for treatment; grade 2, meaning any deviation with the need for pharmacological treatment; grade 3, deviations requiring surgical, endoscopic, or radiological intervention, divided into 3-a, not under general anesthesia, and 3-b, under general anesthesia; grade 4, deviations leading to 4-a, single organ disfunction, and 4-b, multi-organ disfunction; to grade 5, meaning death of a patient.

### Statistical analysis

Multiple logistic regression analysis was performed for the intra- and postoperative complications as the dependent variable. Independent variables included in the logistic regression models were intra-abdominal pressure, neuromuscular block, age, gender, BMI, and trial year. Continuous variables were expressed as mean (± standard deviation) and categorical data as number (percentage). All statistical analyses were performed with IBM SPSS Statistics (version 24, Armonk NY).

## Results

### Intra- and postoperative surgical complications

Intra- and postoperative outcomes are presented per randomized group in Tables 2 and 4. Of the 556 living donors, fifty-three (9.5%) patients developed intra-operative complications graded ≥ 2 according to ClassIntra. Chi-square analysis showed *p* values < 0.05 between the groups with different intra-abdominal pressures (group A vs. C: *p* < 0.001, group B vs. D: *p* = 0.009) and between standard IAP with deep NMB compared to low IAP with moderate NMB (group B vs. group C: *p* < 0.001). No significance was present between standard IAP with moderate NMB compared to low IAP with deep NMB (group A vs. group D: *p* = 0.076) (Table 2).

**Table 2:** Intra-operative complications according to ClassIntra grade ≥2 and other intra-operative variables

	A	B	C	D
Intra-abdominal pressure^g^	Standard	Standard	Low	Low
Neuromuscular blockade^h^	Moderate	Deep	Moderate	Deep
	(***n*** = 401)	(***n*** = 78)	(***n*** = 29)	(***n*** = 48)
Intra-operative complications (ClassIntra grade ≥ 2)				
Total number	32 (8.0%)^a,d,e^	2 (2.6%)^a,c,f^	11 (37.9%)^b,c,e^	8 (16.7%)^b,d,f^
Grade 2	30 (7.5%)	2 (2.6%)	10 (34.5%)	8 (16.7%)
Grade 3	2 (0.5%)	0 (0.0%)	0 (0.0%)	0 (0.0%)
Grade 4	0 (0.0%)	0 (0.0%)	1 (3.4%)	0 (0.0%)
Type of complication				
Bleeding	13 (40.6%)	2 (100%)	10 (90.9%)	7 (87.5%)
Organ laceration	16 (50.0%)	0 (0.0%)	1 (9.1%)	1 (12.5%)
Bleeding and organ laceration	2 (6.3%)	0 (0.0%)	0 (0.0%)	0 (0.0%)
Other^i^	1 (3.1%)	0 (0.0%)	0 (0.0%)	0 (0.0%)
Operation time (min)	210.5 (± 73.7)	130.4 (±41.8)	155.2 (±39.9)	119.7 (±33.2)
Estimated blood loss (ml)	62.3 (±69.6)	45.8 (±58.7)	245.7 (±499.8)	76.1 (±128.5)
Conversion to open procedure	2 (3.4%)	1 (1.3%)	4 (13.8%)	3 (6.3%)

Chi-square testing: ^a^A vs B, *p* = 0.088; ^b^C vs D, *p* = 0.109; ^c^B vs C, *p* < 0.001; ^d^A vs D, *p* = 0.076; ^e^A vs C, *p* < 0.001; ^f^B vs D, *p* = 0.009

^g^Standard pressure 12–14 mmHg; low pressure 6–7 mmHg

^h^Moderate blockade: single dose rocuronium or TOF count 1–2; deep blockade: PTC 1–2 or PTC 1–5

^i^Re-laparoscopic procedure for lost gauze (1×)

Multiple logistic regression analysis revealed the use of standard IAP (*OR* 0.318, *95% CI* 0.118–0.862; *p* = 0.024) as a predictor of less intra-operative complications and moderate NMB (*OR* 3.518, *95% CI* 1.244–9.948; *p* = 0.018) as a significant predictor of more intra-operative complications. All graded ≥ 2 according to ClassIntra (Table 3).

**Table 3:** Multiple logistic regression model with intra-operative complications (ClassIntra grade ≥2) and postoperative complications (Clavien-Dindo grade ≥2)

Dependent	Predictors	***B***	***OR*** (***CI***)	***p***
Intra-operative complications (ClassIntra grade ≥ 2)	IAP (standard)	−1.145	0.318 (0.118–0.862)	**0.024**
	NMB (moderate)	1.258	3.518 (1.244–9.948)	**0.018**
	Gender (male)	0.380	1.462 (0.759–2.816)	0.256
	Age	0.024	1.024 (0.997–1.052)	0.085
	BMI	−0.002	0.998 (0.912–1.092)	0.963
	Trial year	0.217	1.242 (0.602–2.562)	0.558
Postoperative complications (Clavien-Dindo grade ≥ 2)	IAP (standard)	0.493	1.638 (0.513–5.234)	0.405
	NMB (moderate)	−0.446	0.640 (0.274–1.498)	0.304
	Gender (male)	0.198	1.219 (0.606–2.450)	0.579
	Age	0.005	1.005 (0.976–1.035)	0.747
	BMI	−0.037	0.964 (0.870–1.068)	0.484
	Trial year	0.684	1.982 (0.828–4.744)	0.124

Thirty-one (6.8%) postoperative complications with grade ≥ 2 according to the Clavien-Dindo classification were noted (Table 4). Chi-square analysis and multiple logistic regression analysis did not show a significant association between IAP or NMB as independent predictors of postoperative complications (Tables 3 and 4).

**Table 4:** Postoperative complications (Clavien-Dindo grade ≥ 2) and hospital admission

	A	B	C	D
Intra-abdominal pressure^**g**^	Standard	Standard	Low	Low
Neuromuscular blockade^**h**^	Moderate	Deep	Moderate	Deep
	(***n*** = 298)	(***n*** = 78)	(***n*** = 29)	(***n*** = 48)
Postoperative complications (Clavien-Dindo grade ≥ 2)				
Total number	17 (5.7%)^a,d,e^	9 (11.5%)^a,c,f^	2 (6.9%)^b,c,e^	3 (6.3%)^b,d,f^
Grade 2	17 (5.7%)	9 (11.5%)	2 (6.9%)	2 (4.2%)
Grade 3	0 (0.0%)	0 (0.0%)	0 (0.0%)	1 (2.1%)
Type of complication				
Infection	8 (47.1%)	7 (77.8%)	0 (0.0%)	0 (0.0%)
Bleeding	3 (17.6%)	0 (0.0%)	0 (0.0%)	0 (0.0%)
Ileus/gastroparesis	2 (11.8%)	1 (11.1%)	0 (0.0%)	1 (33.3%)
Other^i^	4 (23.5%)	1 (11.1%)	2 (100%)	2 (66.7%)
Length of hospital admission (days)	4.0 (±0.9)	3.7 (±1.0)	4.7 (±1.3)	3.9 (±1.3)

Chi square testing: ^a^A vs B, *p* = 0.097; ^b^C vs D, *p* = 0.917; ^c^B vs C, *p* = 0.522; ^d^A vs D, *p* = 0.887; ^e^A vs C, *p* = 0.806; ^f^B vs D, *p* = 0.369

^g^Standard pressure 12–14 mmHg; low pressure 6–7 mmHg

^h^Moderate blockade: single dose rocuronium or TOF count 1–2; deep blockade: PTC 1–2 or PTC 1–5

^i^Hypertension (3×), atrial fibrillation/supraventricular tachycardia (2×), pneumothorax (1×), meatus stenosis (1×), respiratory insufficiency, no diagnosis reported (1×), subcutaneous emphysema (1×)

Logistic regression analysis did not show a significant association between intra-operative complications, graded ≥2 according to ClassIntra, and postoperative complications according to Clavien-Dindo classification graded ≥2 (*OR* 0.181, *95% CI* 0.407–3.532; *p* = 0.742).

### Other outcomes

Linear regression analysis on other outcomes is presented in Table 5. The estimated blood loss was significantly lower with standard IAP and higher with moderate NMB as well as within males (resp. *β* 0.240, *p* = 0.000; *β* −0.219, *p* = 0.000; and *β* −0.088, *p* = 0.034). With standard IAP, moderate NMB, and within males, operation time was significantly longer (resp. *β* −0.074, *p* = 0.049; *β* −0.236, *p* = 0.000; and *β* −0.106, *p* = 0.002). Trial year was a significant predictor of operation time (*β* −0.419; *p*=0.000). The length of hospital admission was also significantly shorter in the trials conducted in more recent years (resp. *β* −0.157; *p* = 0.007).

**Table 5:** Multiple linear regression on other outcomes

Dependent	Predictors	***B***	Beta coefficient (***CI***)	***p***
Estimated blood loss	IAP (standard)	87.499	0.240 (54.694–120.304)	**0.000**
	NMB (moderate)	−66.039	−0.219 (−94.590 to −37.488)	**0.000**
	Gender (male)	−22.375	−0.088 (−43.047 to −1.703)	**0.034**
	Age	0.338	0.032 (−0.523–1.198)	0.441
	BMI	−0.232	−0.007 (−3.088–2.625)	0.874
	Trial year	3.505	0.014 (−20.464–27.474)	0.774
Operation time	IAP (standard)	−16.042	−0.074 (−32.045 to −0.039)	**0.049**
	NMB (moderate)	−42.115	−0.236 (−56.042 to −28.188)	**0.000**
	Gender (male)	−15.932	−0.106 (−26.016 to −5.848)	**0.002**
	Age	0.045	0.007 (−0.375–0.465)	0.834
	BMI	0.577	0.028 (−0.816–1.971)	0.416
	Trial year	−63.773	−0.419 (−75.465 to −52.081)	**0.000**
Length of hospital admission	IAP (standard)	−0.052	−0.021 (−0.485–0.381)	0.812
	NMB (moderate)	−0.280	−0.128 (−0.610–0.050)	0.096
	Gender (male)	0.070	0.033 (−0.240–0.379)	0.658
	Age	0.004	0.041 (−0.010–0.019)	0.584
	BMI	−0.015	−0.044 (−0.066–0.035)	0.549
	Trial year	−0.157	−0.237 (−0.270 to −0.043)	**0.007**

## Discussion

This analysis shows an incidence of fifty-three (9.5%) intra-operative complications grade ≥2 according to ClassIntra(Dell-Kuster et al., 2020) in 556 patients undergoing laparoscopic donor nephrectomy. Deep NMB was a significant predictor for less intra-operative complications. Four of the listed studies investigated the relationship between the depth of NMB and/or IAP, clinical outcomes, and intra-operative complications in laparoscopic donor nephrectomy (LDN). One study indicates that the use of low pressure could lead to lower pain scores and a better recovery after LDN(Warlé et al., 2013), but this could not be confirmed by another trial.(Özdemir-van Brunschot et al., 2017) Moreover, it has been shown that the use of low pressure with moderate NMB may compromise safety,(Özdemir-van Brunschot et al., 2018) where two studies indicate that the use of a deep NMB improves intra-operative safety during low and standard pressure LDN.(Özdemir-van Brunschot et al., 2018; Bruintjes et al., 2019) An earlier meta-analysis(Bruintjes et al., 2017) showed the surgical working field was significantly improved during laparoscopic surgeries facilitated by a deep NMB as compared with a moderate NMB. Therefore, a lower incidence of surgical complications during laparoscopic surgery with a deep NMB is in line with existing literature.(Torensma et al., 2016; Bruintjes et al., 2017; Özdemir-van Brunschot et al., 2018; Martini et al., 2014; Kim et al., 2016; Koo et al., 2016; Yoo et al., 2015; Dubois et al., 2014; Staehr-Rye et al., 2014) Additionally, a recent randomized controlled trial in patients undergoing gastric bypass surgery showed that poor surgical conditions were associated with a higher incidence of intra-operative surgical complications (61.5% in the moderate block versus 15.3% in the deep NMB block; *p*  < 0.001).(Fuchs-Buder et al., 2019) These findings support the beneficial influence of a deep NMB on the risk of intra-operative complications. Maintaining a deep NMB until the end of surgery challenges the anesthetic team, since adequate neuromuscular monitoring is required, which can be challenging if the arms cannot be positioned in abduction. Moreover, additional training may be required for adequate continuous perfusion of a neuromuscular blocking agent, dose adjustments and the use of antagonizing agents (i.e. sugammadex) to prevent residual NMB. Preventing residual NMB decreases the risk of postoperative pulmonary complications (Cammu, 2020). Furthermore, the costs related to the use of antagonizing agents can be a hurdle in routine practice. Given these challenges, a higher level of evidence is warranted, necessitating a prospective randomized clinical trial to confirm the hypothesis that the use of deep NMB reduces intra-operative complications, thereby improving patient safety.

Although consensus guidelines from the Dutch and European Societies of Endoscopic Surgery(Neudecker et al., 2002; la Chapelle et al., 2012) state that the lowest possible IAP with an adequate surgical field should be used, a vast majority of laparoscopic surgeons use a routine insufflation pressure of ≥ 12 mmHg. Probably, the main reason for this is that the use of low IAP (<10 mmHg) may hamper the quality of the surgical field. Our data confirm that low IAP was not associated with an increased risk of intra-operative complications. This indicates that the stepwise increase of IAP in case of inadequate surgical conditions is a safe approach to apply low IAP if possible, during LDN. Therefore, our data support the above-mentioned guidelines regarding the use of a low IAP.

In this study, we found no relationship between intra- and postoperative complications, which is not fully in line with findings in earlier published trials. Bohnen et al*.(Bohnen et al.,* 2017*)* showed, among patients undergoing abdominal surgery, that intra-operative complications were independently associated with an approximately 3-fold increase in 30-day postoperative complications and increased length of hospital admission.(Bohnen et al., 2017; Hu et al., 2012; Boon et al., 2018) A recent large validation study of the IntraClass by Dell-Kuster et al. among 2520 patients undergoing any type of surgery clearly demonstrated a strong correlation between intra- and postoperative complications (Dell-Kuster et al., 2020), especially more severe intra-operative complications graded 3 and 4 correlated with postoperative complications. In the studies included for this pooled analysis, only grade 2 intra-operative complications were observed. This may very well explain why we could not find this relationship between intra- and postoperative complications. With cumulating evidence indicating an association between intra-operative complications and 30-day postoperative mortality, postoperative morbidity, and length of hospital admission,(Kinaci et al., 2016; Dell-Kuster et al., 2020; Bohnen et al., 2017; Hu et al., 2012; Boon et al., 2018) a prospective, randomized trial is required to establish a possible relationship between the use of deep NMB and a lower incidence of postoperative complications after laparoscopic surgery.

A strength of this study is the inclusion of relatively healthy individuals undergoing a highly standardized surgical procedure, performed in academic teaching hospitals throughout the Netherlands. This contributed to a relatively high internal validity of the trials used for the pooled analysis. All ClassIntra grade ≥2 intra-operative complications were prospectively recorded by a double-blinded observer which reduces the risk of observer bias. Moreover, our pooled analysis of individual patient data allowed us to perform multiple variable regression analyses to identify independent predictors of intra-operative complications.

The retrospective nature of the pooled, individual patient, data analysis is a limitation of this study. These trials were not powered or designed to study the effect of NMB or IAP on the risk of intra-operative complications. Therefore, a certain degree of confounding bias cannot be ruled out. Another limitation of this study is the intra-operative complications were graded retrospectively by three blinded researchers (EH, GRB, and MW) according to the ClassIntra classification for intra-operative complications. This classification was proposed by Dell-Kuster et al.(Dell-Kuster et al., 2020) and was developed to grade all patient-related intra-operative complications including all deviations from the ideal intra-operative course.(Dell-Kuster et al., 2015; Dell-Kuster et al., 2020) An underreporting of small complications with intervention is understandable, and minor deviations from the ideal course without the need for an additional intervention (ClassIntra grade 1) were not actively recorded. The low event rate is another limitation of this study which leads to the need for large randomized clinical trials with a higher event rate to confirm our findings. Moreover, the included studies vary from 2001 to 2017. The learning curve not only of individual surgeons, but also of the whole team (or center) could have been a potential source of bias.

In conclusion, our data indicate that the use of a deep NMB increases safety during laparoscopic donor nephrectomy, when compared with moderate NMB. The use of low IAP with a stepwise increase in case of inadequate surgical conditions was not associated with an increased risk of intra-operative complications and may therefore be a safe strategy for using lower insufflation pressures. The EURO-relax trial(GHM et al., 2021) will reveal if the routine use of deep NMB throughout laparoscopic surgery decreases intra- and postoperative complications and thereby improves patient safety.

## Data Availability

The pooled dataset analyzed during this study is available from the corresponding author on reasonable request.

## Abbreviations

IAP: Intra-abdominal pressure
NMB: Neuromuscular blockade
TOF: Train of four
PTC: Post-tetanic count
